# *Fomitopsis meliae* CFA 2, a novel brown rot for endoglucanase: emphasis towards enhanced endoglucanase production by statistical approach

**DOI:** 10.1080/21501203.2021.1918277

**Published:** 2021-04-30

**Authors:** Amisha Patel, Jyoti Divecha, Amita Shah

**Affiliations:** a P. G. Department of Biosciences, Sardar Patel University, Satellite Campus, Anand, Gujarat, India; b Department of Statistics, Sardar Patel University, Gujarat, India

**Keywords:** Brown rot, *Fomitopsis meliae* CFA 2, endoglucanase, response surface methodology

## Abstract

Brown rot basidiomycetes are a principal group of wood-decaying fungi which degrade wood cellulose and hemicellulose by the combination of carbohydrate active enzymes and non-enzymatic oxidation reactions. Very scant information is available on carbohydrate active enzymes of brown rot fungi. In this context, present study focused on the production of cellulolytic–hemicellulolytic enzymes from newly isolated brown rot *Fomitopsis meliae* CFA 2. Under solid-state fermentation using wheat bran as the substrate *Fomitopsis meliae* CFA 2 was able to produce a maximum of 1391.12 ± 21.13 U/g of endoglucanase along with other cellulolytic and hemicellulolytic enzymes. Various fermentation parameters were optimised for enhanced production of endoglucanase by employing Plackett-Burman design followed by Box-Behnken design. A well-fitted regression equation with R^2^ value of 98.91% was attained for endoglucanase. The yield of endoglucanase was enhanced by 1.83-fold after executing statistical optimisation of various fermentative parameters. The newly isolated *Fomitopsis meliae* CFA 2 was found to be a potential producer of endoglucanase. Enzymatic saccharification of alkali-treated wheat straw and rice straw resulted in release of 190.8 and 318.8 mg/g of reducing sugars, respectively.

## Introduction

Cellulose is the most abundant polysaccharide in the plant cell wall. It is a linear polymer composed of varying number of *β*-D-glucopyranose residues, linked by (1→4) glycosidic bonds (Da Silva et al. 2016). Cellulase is a complex of enzymes that work synergistically to attack on native cellulose. Cellulase is a family of at least three groups of enzymes: Endoglucanases (EC 3.2.1.4) or β-1,4-D-glucan-4-glucanohydrolases, cut the internal glycosidic linkages in amorphous cellulose randomly and generate oligosaccharides of various chain lengths and consequently open new chain ends; Exoglucanases, including β-1,4-D-glucan glucanohydrolases (also known as cellodextrinases) (EC 3.2.1.74) and β-1,4-D-glucan cellobiohydrolases EC 3.2.1.91) act in a processive manner on reducing or nonreducing ends of cellulose polysaccharide chains, releasing either glucose (glucanohydrolases) or cellobiose (cellobiohydrolase) as major products; β-D-Glucosidases, or β-D-glucosideglucohydrolases (EC 3.2.1.21), act on cellobiose and cellodextrins and release D-glucose units (Shah et al. 2017).

In the present scenario, fungal cellulases have gained immense attention of researcher and industries due to their enormous biotechnological applications mainly in biomass refining, food, animal feed, paper and pulp industries (Kuhad et al. 2011). The major bottleneck for application of cellulases is the high cost of enzyme production. Efforts towards cost reduction have been directed in a search for stable enzymes from hyperactive microbial strains and efficient fermentation techniques (Xu et al. 2005). The use of solid state fermentation (SSF) using cheap biomass as the substrate can improve production economics (Wen et al. 2005).

Wood decay is a vital process in nature, as huge amounts of fixed carbon from wood, which is released as greenhouse gas during the process of decay. Although many types of organisms can degrade wood, the most important and potent are the wood-rotting fungi (Eriksson et al. 2012). They are basically classified into three categories according to their wood degradation pattern, that is, white, soft and brown-rot fungi. White rot and soft rot have been extensively studied while brown rots have not been studied much. Cellulolytic enzymes from white rot and soft rot fungi have been extensively studied in model organisms such as *Phanerochaete chrysosporium* and*Trichoderma viride*, respectively. Brown rot fungi rapidly utilise plant cell wall polysaccharides, namely, cellulose and hemicellulose without removing lignin. However, little information is available on cellulolytic enzymes of this group of fungi. With respect to the pattern of cellulose degradation and cellulolytic enzymes produced, the brown rot fungi differ substantially from soft rot and white rot fungi. Brown rot are generally reported to lack the exoglucanases that can hydrolyse crystalline cellulose (Kuhad et al. 1997), yet they cause the most destructive type of wood decay and are important contributors to biomass recycling. The wood decay by brown-rot fungi involves the hydroxyl radical-mediated oxidation of lignocellulosic biomass via Fenton reaction, followed by the enzymatic degradation of cellulose and hemicellulose (Shah et al. 2018). Very few reports are available on cellulase production from brown rot fungi (Deswal et al. 2011; Wang et al. 2012; Park et al. 2015).

*Fomitopsis* is one of the most prominent wood-decaying genus of basidiomycetes fungus which cause brown rot mostly in softwood and in some species of hardwood. Very few cellulolytic and hemicellulolytic enzymes of this fungus have been studied, including endoglucanases from *Fomitopsis palustris* (Yoon and Kim 2005; Shimokawa et al. 2008; Song et al. 2008; Cha et al. 2018) and *Fomitopsis pinicola* (Yoon et al. 2008); β-glucosidases from *Fomitopsis palustris* (Yoon and Kim 2005; Yoon et al. 2008; Ji and Cha 2010; Okamoto et al. 2011) and *Fomitopsis pinicola* (Joo et al. 2009; Park et al. 2015); cellobiohydrolases from *Fomitopsis palustris* (Yoon and Kim 2005); processive endoglucanases from *Fomitopsis palustris* (Yoon et al. 2007) and xyalanase from *Fomitopsis pinicola* (Lee et al. 2010). The fungus was also reported for its efficient utilisation in the bioconversion of lignocellulosic biomass to fermentable sugars (Yoon and Kim 2005; Shimokawa et al. 2008; Deswal et al. 2011; Wang et al. 2012) and the subsequent fermentation of sugars to ethanol (Okamoto et al. 2011). Moreover, statistical optimisation of cellulase production was reported for *Fomitopsis palustris*(Wang et al. 2012). Overall, the enzyme profiles are available only for two species of *Fomitopsis* genus.

In view of above facts, the present work was focused on the optimisation of endoglucanase production by a newly isolated brown rot fungus under solid state fermentation (SSF) using response surface methodology. Efficacy of multi-enzyme cocktail produced by SSF was also checked in saccharification of biomass.

## Materials and methods

### Substrate, chemicals and media

Wheat bran used for enzyme production was procured from a local market; wheat straw and rice straw were procured from local farmer whereas sugarcane bagasse was procured from sugar industry. All the agro residues were washed thoroughly with water, dried at 80°C and stored at room temperature in airtight plastic bags until use. *p*-Nitrophenyl-α-L-arabinofuranoside (PNAF), *p*-nitrophenyl-β-D-xylopyranoside (PNXP), *p-*nitrophenyl-β-D-glucopyranoside (PNGP), *p*-nitrophenyl-β-D-cellobioside (PNCB), carboxymethyl-cellulose (CMC), Avicel PH-101, birch wood xylan and cellobiose were purchased from Sigma (St. Louis, MO, USA). Commercial cellulase SIGMA (Cellulases from *T. reseei* ATCC 26,921, ≥700 U/g) was purchased from Sigma-Aldrich, USA. All the chemicals, reagents and media used in the present study were of analytical grade purchased from Qualigens, Hi-media, Merck, Loba from India.

### Strain isolation and identification

Screening for cellulase-producing microorganisms was carried out from various natural samples including soil samples, decaying waste materials. Soil samples were serially diluted whereas other samples were surface sterilised and then plated on potato dextrose agar (PDA) medium. The purified isolates were checked for cellulose, xylan and mannan hydrolysis on a medium containing the following composition (g/l): ammonium tartrate 5.0; KH_2_PO_4_1.0; MgSO_4_ · 7H_2_O 0.5; yeast extract 0.1; CaCl_2_ · 7H_2_O 0.001; agar 2.5% (w/v) and pH 5.5 (Medeiros et al. 2003). Carboxy methyl cellulose (CMC), Avicel pH 101, birchwood xylan, and glucomannan were added as carbon source individually at the rate of 1% for screening of cellulolytic and hemicelluloytic activity. The plates were incubated at 30°C for 72 h. After incubation zone of clearance was revealed using 0.1% congored solution. Fungal cultures showing significant zone of hydrolysis were selected, purified and maintained on PDA for further experiments. Molecular identification was carried out at Eurrofins India Pvt Ltd, Bangalore. Sequence data were submitted to the NCBI (National Centre for Biotechnology Information).

ITS gene sequences of fungi belong to the genus *Fomitopsis* species were downloaded from GenBank in FASTA format. Sequences were analysed and edited by using BioEdit 7.2.5 (Hall 1999). To find out the common regions among all retrieved *Fomitopsis* species sequences, pairwise alignment and multiple sequence alignment (MSA) was carried out using Clustal-W (Thompson et al. 2003) embedded in MEGA 7.0 (Tamura et al. 2013). All positions having gaps and missing data were eliminated. Phylogenetic analyses were performed using maximum likelihood (ML) approach in RaxMLGUI v. 1.5b1 (Silvestro and Michalak 2012). An ML analysis was run for 1000 bootstrap replicates under the GTR + I model to assess clade support.

### Enzyme production under solid-state fermentation

Solid state fermentation was carried out in 250 ml Erlenmeyer flasks, each having 5.0 g of dry agro residues (wheat bran, wheat straw, rice straw, and sugarcane bagasse) moistened with mineral salt solution (g/l): (NH4)_2_SO_4_, 0.5; KH_2_PO_4_, 0.5; MgSO_4_, 0.5; Urea, 0.5, tween 80 0.2%, and pH 5.5 to attain the final substrate-to-moisture ratio of 1:3.5. The flasks were sterilised by autoclaving at 121°C (15 psi), and thereafter cooled to room temperature and inoculated with five mycelial discs (9 mm). The contents of the flasks were mixed well and incubated at 30°C.

### Enzyme extraction

The contents from each flask were extracted using 30ml citrate buffer (50 mM, pH 4.8) along with 0.2 ml of Tween-80. The contents of flasks were mixed on shaker (150 rpm for 30 min) at 30°C, and filtered through a wet muslin cloth thorough squeezing. The centrifugation of filtrate was carried out at 8000 rpm for 30 min. The clear supernatant thus recovered was utilised as a crude enzyme for further studies.

### Enzyme assays

Endoglucanase assay was carried out using 2% CMC in 50 mM sodium citrate buffer, pH 4.8 as substrate. The release of reducing sugars in 30 min at 55°C was determined by the dinitrosalicylic acid (DNS) method (Miller 1959). Filter paper activity (FPase) was measured using filter paper (Whatman no. 1) as substrate (Ghosh 1994). The release of reducing sugars was measured in 60 min at 50°C and pH 4.8 (50 mM sodium citrate buffer) using DNS method. One unit of endoglucanase/filter paper activity is defined as amount of enzyme releasing 1 μM of glucose per minute under assay conditions. Xylanase assay was carried out using 1% birch wood xylan solution in 50 mM sodium citrate buffer (pH 5.3) (Bailey et al. 1992) at 50°C for 10 min. Mannanase assay was carried out using 0.5% locust bean gum (LBG) solution in 50 mM sodium citrate buffer (pH 5.3) at 50°C for 10 min. The enzyme reaction was stopped by addition of 1 ml DNS reagent (Miller 1959). One unit of xylanase/mannanase activity is defined as quantity of enzyme required to liberate 1 μM of xylose/mannose per minute under assay condition. Cellobiohydrolase activity was determined using 1 mM p-nitrophenyl-β-D-cellobioside (PNCB) as a substrate at 50°C and pH 4.8 (50 mM sodium citrate buffer) for 20 min (Tuohy et al. 2002). β-glucosidase assay was carried out using 2 mM p-nitrophenyl-β-D-glucopyranoside (PNGP) as a substrate at 55°C and pH 4.8 (50 mM sodium citrate buffer) for 30 min. β-xylosidase assay was carried out using 2 mM *p*-nitrophenyl-β-D-xylopyranoside (PNXP) as a substrate at 65°C and pH 4.0 (50 mM sodium citrate buffer) for 30 min. α-L-Arabinofuranosidase assay was carried out using 1 mM *p*-nitrophenyl- α-L-arabinofuranoside (PNAF) as a substrate at 55°C and pH 4.0 (50 mM sodium citrate buffer) for 10 min. One millilitre of 2 M sodium carbonate was used to terminate the enzyme reaction. The absorbance was measured at 410 nm to determine the amount of *p*-nitrophenol released. One unit of cellobiohydrolase/β-glucosidase/β-xylosidase/α-L-arabinofuranosidase activity is defined as amount of enzyme required to release 1 μM of *p*-nitrophenol per minute under assay condition (Patel et al. 2016).

### Optimisation of endoglucanase production using response surface methodology

Optimisation of physicochemical parameters for endoglucanase production was performed in two stages. Initially, four variables were considered for screening using Plackett-Burman Design (PBD) to identify the variables, which significantly influenced endoglucanase production and in the second stage, the significant variables were optimised using a Box-Behnken Design (BBD).

### Screening of significant parameters by PBD

In present study, pH, moisture ratio, fermentation time and inoculum were selected as the independent variables. Each variable was set at two levels, high and low (Table 1). The experimental design is given in Table 2. The significance of regression coefficients for endoglucanase was tested by *t*-test.

**Table 1.** Actual and coded level of variables tested with PBD for endoglucanase production

Process variables	Code	−1	1
pH	A	3	7
Moisture ratio	B	1:1	1:5
Inoculum (mycelial disc)	C	1	7
Fermentation time (h)	D	72	240

**Table 2.** PBD matrix for the screening of variables influencing endoglucanase production

Run	pH	Moisture ratio	Inoculum	Fermentation time (h)	Endoglucanase (U/g)
1	7	1:1	1	240	114.70
2	7	1:5	1	72	2.05
3	7	1:5	7	72	14.06
4	3	1:5	7	240	481.90
5	7	1:1	7	240	215.90
6	3	1:5	1	240	486.80
7	3	1:1	7	72	18.17
8	3	1:1	1	72	4.08

**Table 3.** Regression Coefficient for endoglucanase production

Term	Coef	SE Coef	*t*-Value	*p*-Value
Constant	167.2	13.3	12.61	0.006
pH	−80.5	13.3	−6.07	0.026
Moisture ratio	79.0	13.3	5.96	0.027
Inoculum	15.3	13.3	1.15	0.368
Fermentation time (h)	157.6	13.3	11.88	0.007
Inoculum* Fermentation time (h)	8.8	13.3	0.66	0.576

### Optimisation of significant parameters for endoglucanase production by BBD

BBD, a three-level response surface design (with a total of 15 experimental runs) was used for the optimisation of endoglucanase production (Table 5). The design allowed us to evaluate the main and interactive effects of independent variables pH (X1), moisture ratio (X2) and fermentation time (X3) on endoglucanase activity (U/g). Endoglucanase activity (U/g) corresponding to the combined effects of three variables was studied in their specific ranges as shown in Table 4. The temperature was set at 30°C during the entire experiment. All the flasks were analysed for endoglucanase activity at specific time intervals as planned in BBD. BBD was used because it is a good response surface design for fitting a quadratic model comprising linear, quadratic and interaction effect terms. Quadratic/second order models considered as response surface models for predicting the optimal points are expressed according to Equations (1) and (2). For statistical computations the independent variables were coded as:

(1) $$x_{i}=\;\left(X_{i}-X_{0}\right)/\delta X_{i}$$

**Table 4.** Actual and coded level of variables tested with BBD for endoglucanase production

Process variables	Coded level of variables		
	−1	0	1
pH (X1)	3	5	7
Moisture ratio (X2)	1:1	1:3	1:5
Fermentation time (h) (X3)	72	156	240

**Table 5.** Full factorial BBD matrix for endoglucanase production

Runs	X1	X2	X3	Endoglucanase	
				Predicted activity (U/g)	Experimental activity (U/g)
1	−1	−1	0	91.77	83.10
2	−1	1	0	335.70	356.31
3	1	−1	0	82.46	77.70
4	1	1	0	403.38	445.50
5	−1	0	−1	34.17	32.20
6	−1	0	1	853.83	942.97
7	1	0	−1	35.63	32.27
8	1	0	1	884.11	938.38
9	0	−1	−1	6.61	8.50
10	0	−1	1	696.34	410.41
11	0	1	−1	6.61	6.03
12	0	1	1	696.34	954.63
13	0	0	0	486.60	485.44
14	0	0	0	486.60	489.41
15	0	0	0	486.60	484.99

**Table 6.** Estimated regression coefficients for ln(EndoU/g)

Term	Coef	SE Coef	*t*-Value	*p*-Value
Constant	6.18746	0.15807	39.143	0.000
pH	0.01919	0.09680	0.198	0.849
Moisture ratio	0.72109	0.11177	6.451	0.001
Fermentation time (h)	1.60734	0.11177	14.380	0.000
pH*pH	−0.02904	0.14248	−0.204	0.845
Moisture ratio*Moisture ratio	−0.97145	0.14248	−6.818	0.000
Fermentation time (h)* Fermentation time (h)	−0.99860	0.14248	−7.008	0.000
pH*Moisture ratio	0.07265	0.13689	0.531	0.615
pH*Fermentation time (h)	−0.00176	0.13689	−0.013	0.990

R-Sq = 98.91% R-Sq(pred) = 94.19% R-Sq(adj) = 97.46%

where *x_i_* is the experimental coded value of the variable; *X_0_* is the middle value of *X_i_* and *δX_j_* is the step change for *i = *1, 2, 3. Thus the independent variables were taken at a central coded value considered as zero.

Endoglucanase production (response *Y*) was explained as a second-order response model on three independent variables given by

(2) $$Y\;=\;\beta_{0}+\;\Sigma \;\beta_{i}x_{i}+\;\Sigma \;\beta_{ii}{x_{i}}^{2}+\;\Sigma \beta_{ij}x_{i}x_{j}$$

where *Y* is the predicted response variable, *β_0_, β_i_, β_ii_, β_ij_* are fixed regression coefficients of the model representing the constant, linear, quadratic and interaction effects, respectively, of the independent variables, *x_i_, x_j_* (*i* = 1,2,3, *i ≠ j*) represents independent variables in the form of coded values.

### Interpretation and data analysis

The results of both the experimental designs were analysed and interpreted using MINITAB 16 (PA, USA) statistical software. It consists of statistical estimation and testing of the regression coefficients. The statistical model is fitted in terms of coded values of the independent variable for easy interpretation, but for a user-friendly graphical view it is estimated in terms of original values of experimental factors. Analysis of variance (ANOVA) is used to establish the significance of the model regression coefficients and the insignificance of the lack of fit of the model. When the adjusted R^2^ and predicted R^2^ of a model are within 0.20 of each other, this means that the fitted response surface model in an adequate number of terms is suitable for prediction of the optimum fermentation parameters, and a response contour plot is made showcasing the model. Finally, the optimum combination of experimental factors is read from the contour plot for individual responses and from the overlaid plot for the dual responses (Myers et al. 2016).

### Effect of temperature and pH on endoglucanase activity

The optimum temperature for endoglucanase was determined by assaying relative activity at different temperatures ranging from 40°C to 90°C. The optimum pH for endoglucanase was determined by assaying relative activity at different pH (3.0–7.0) using 50 mM sodium citrate buffer for pH 3.0, 4.0, 4.8, 5.0 and 50 mM sodium phosphate buffer for pH 6.0 and 7.0.

### Effect of temperature on endoglucanase stability

To determine the thermal stability of endoglucanase, the enzyme solution was treated at different temperatures (50, 60 and 70°C) in 50 mM sodium citrate buffer (pH 4.8) in a temperature-controlled water bath and the residual activity was measured at different time intervals up to 3 h.

### Evaluation of crude enzyme for enzymatic saccharification of agro-residues

#### Pretreatment of wheat straw and rice straw

Pretreatment of wheat straw was given by suspending wheat straw in 5% aqueous NaOH solution (1:10 w/v) and heated in microwave oven (LG Electronics Co., Ltd.; Model no MC3283PMPG) for 20 min. Pretreatment of rice straw was given by suspending rice straw in 5% aqueous NaOH solution (1:10 w/v) and heated at 121°C temperature, 15 lbs for 30 min. The solid residues after each pretreatment were collected by filtration using wet muslin cloth, washed with tap water until neutralisation and dried at 80°C. Cellulose, hemicellulose and lignin contents of the untreated and MAA treated WS were estimated using method developed by Goering and Van Soest (1970).

#### Enzymatic saccharification of pretreated agro-residues

Ultrafiltration of crude enzyme was carried out using Amicon Ultra 3.0 kDa-cut off membrane (Millipore) to remove residual sugars. Enzymatic saccharification of alkali-treated agro residues (wheat straw and rice straw) were carried out using 5 FPU/g of crude enzyme from *Fomitopsis meliae* CFA 2, 5 FPU/g of crude enzyme from *Aspergillus niger* ADH-11 and 5 FPU/g of commercial cellulase from SIGMA. Crude enzyme from *Aspergillus niger* ADH 11 was produced using previously optimised enzyme conditions under solid state fermentation (Patel et al. 2017). Enzymatic saccharification was performed in 150-ml screw cap Erlenmeyer flasks containing 2.5% alkali-treated ago residues in 50 mM sodium citrate buffer (pH 4.8) containing 10 mg% sodium azide and 0.1% Tween-80 (v/v). Saccharification was carried out at 50°C temperature in a temperature controlled shaking water bath. Samples were withdrawn at regular intervals, centrifuged at 8000 rpm for 10 min and the supernatant was analysed for reducing sugars released by DNS method (Miller 1959).

#### Analytical methods

Reducing sugars were analysed by the dinitrosalysilic acid method (Miller 1959). Oxalic acid was analysed using a high-performance liquid chromatography system (Shimadzu, Japan) equipped with a refractive index detector (RID) and a Phenomenex, Rezex ROA-Organic acid H^+^(8%) column with dimensions of 300 mm × 7.8 mm. Samples were eluted using 5.0 mM H_2_SO_4_ with a flow rate of 0.6 ml/min. Column temperature was kept at 50°C throughout the analysis. Fe^3+^ -reduction assay was carried out using ferrozine assay method (Shah et al. 2018). A reaction mixture was contained 50 μl of crude extract, 1.0 ml of 0.1 M acetate buffer (pH 4.5), 25 μl of 1.0 mM FeCl_3_ and 20 μl of 1% (w/v) ferrozine (Sigma Aldrich). The reaction mixture was incubated in the dark for 5 min at room temperature. Fe^3+^ -reducing activity was analysed by measuring absorbance at 562 nm. A standard curve was created using varying concentrations of FeSO_4_.

## Results and discussion

### Isolation and identification of cellulose-degrading fungus

**Figure 1.**
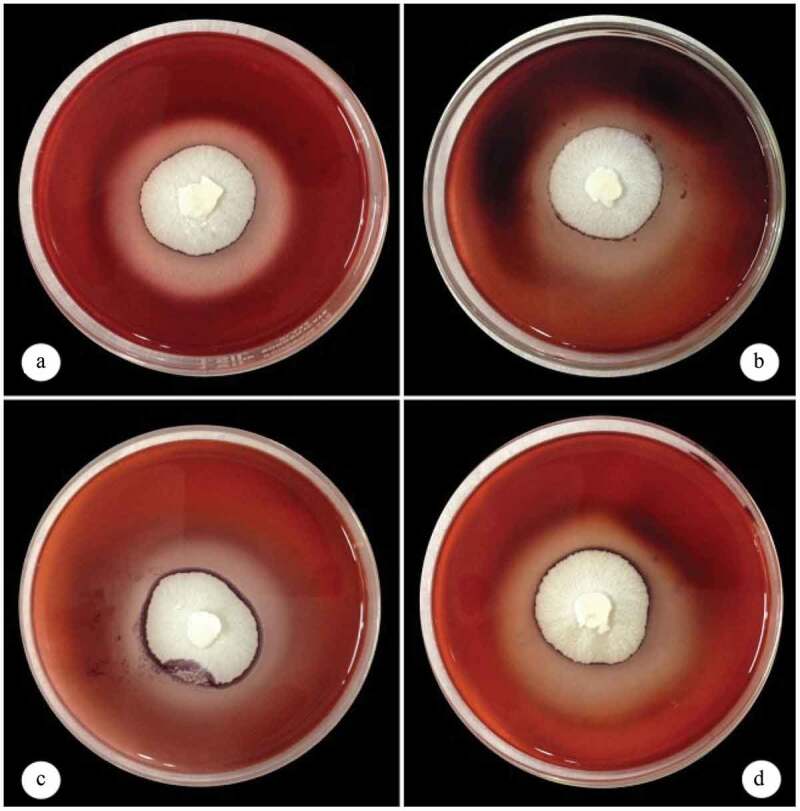
*Fomitopsis meliae* CFA 2 showing zone of hydrolysis on medium containing A) carboxy methyl cellulose B) locust bean gum (Mannan) C) microcrystalline cellulose and D) birch wood xylan

**Figure 2.**
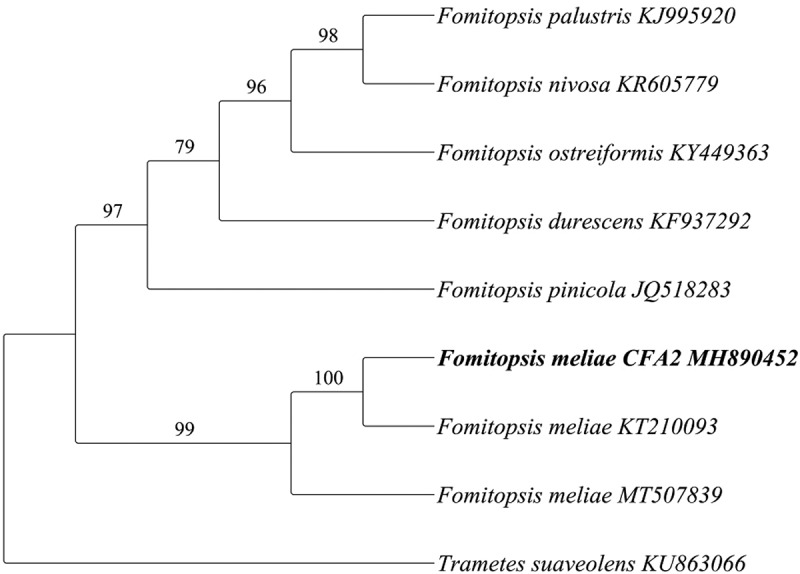
Maximum likelihood cladogram generated from ITS dataset for *Fomitopsis meliae* and other species. Bootstrap values are indicated on the tree branches

Screening of cellulolytic microorganisms was carried out from soil and degraded plant materials. The celluloytic strain used in the present study was isolated from degraded plant material. The newly isolated fungal strain CFA 2 showed a clear zone on CMC, xylan, mannan and microcrystalline cellulose (Avicel) agar plates (Figure 1) and relative zone of hydrolysis were found to be 1.68 ± 0.02, 1.86 ± 0.04, 1.67 ± 0.01, and 1.62 ± 0.04, respectively. The fungal isolate CFA 2 was identified based on the sequence variation present in ITS (internal transcribe spacer) region. Sequence analysis suggested that CFA 2 was phylogenetically related to the members of genus *Fomitopsis* where it showed maximum similarity with *Fomitopsis meliae* (Figure 2). Based on sequence homology and phylogenetic analysis, the strain was identified as *Fomitopsis meliae* CFA 2 (NCBI GenBank Accession Number: MH890452).

*Fomitopsis* P. Karst. was erected in 1881, with *F. pinicola* (Sw.) P. Karst as the type species. The genus belongs to Fomitopsidaceae family of order Polyporales. The genus is characterised by sessile to effused-reflexed basidiomata, a perennial or rarely annual growth habit, either white to tan or pinkish-coloured pore surface having small and regular pores, clamped generative hyphae in a di- to trimitichyphal system, and thin-walled, hyaline, and smooth basidiospores. *Fomitopsis* causes brown rot frequently on gymnosperm trees and sporadically on angiosperm trees (Li et al. 2013). *Fomitopsis meliae* (Underw.) Gilb. was established in 1981 (Gilbertson 1981) and considered as synonyms of *Fomes meliae* (Underw.) Murrill (Murrill 1903) and *Polyporus meliae* Underw (Underwood 1897). *Fomitopsis meliae* has ivory to tan or cinereous pileal surface, and 5–7 per mm sized pores, up to 5 × 10 × 3 cm basidiomata and 6–8 × 2.5–3 μm spores (Li et al. 2013). *Fomitopsis meliae* CFA 2 formed a thick, white and cottony mycelial mat spreading outward from the central inoculum disc after 7 days of incubation on PDA medium.

### Production of endoglucanase under SSF

For SSF, choice of suitable substrate is becoming a very crucial aspect as it anchorages the microbial growth besides serving as source of nutrients and inducers (Shah et al. 2017). Hence, the production of endoglucanase was attempted using various agro residues like wheat bran, wheat straw, rice straw and sugarcane bagasse as a sole carbon source at 30°C under SSF. The yield of endoglucanase was found to be 760.29 ± 11.60 U/g, 65.33 ± 7.32, 78.42 ± 15.66 and 26.91 ± 3.87 using wheat bran, wheat straw, rice straw and sugarcane bagasse, respectively, after 68 h of incubation. It was observed that wheat bran was found to be the most appropriate substrate under SSF, yielding a maximum yield of endoglucanase. Wheat bran acts as a complete nutritious feed for microorganisms having non-starch carbohydrate polymers (~58%), starch (~19%) and crude protein (~18%). The non-starch carbohydrate polymers composed of ~70% of arabinoxylans, ~24% of cellulose and ~6% of β-(1,3) (1,4)-glucan. Wheat bran provides a large surface area as it remains loose even under moist conditions (Sun et al. 2007; Shah et al. 2017).

### Optimisation of endoglucanase production under SSF using response surface methodology

**Figure 3.**
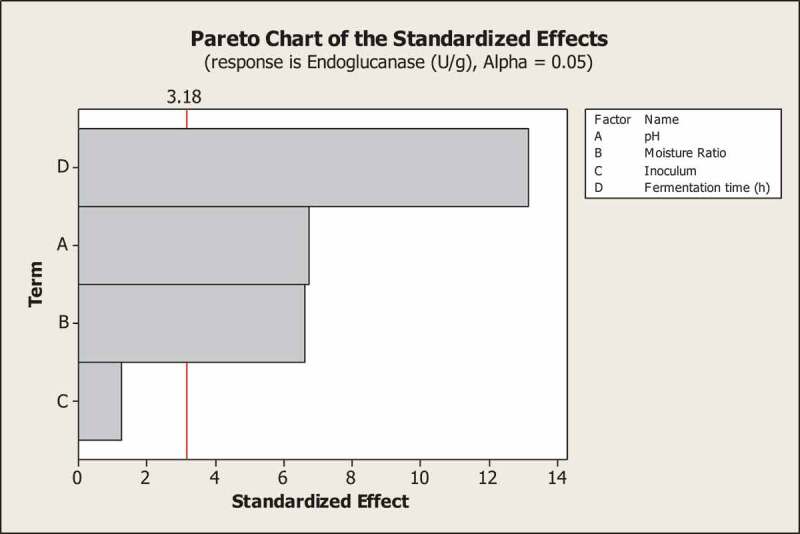
Pareto chart showing effect of media components on endoglucanase production

The aim of the study is to determine the optimum fermentation conditions that enhance the endoglucanase production. Fermentation parameters like incubation time, pH, nitrogen source, moisture content, medium ingredients, inoculum size, surfactant and inducer play a very critical role in enzyme production by fungi under solid state fermentation. Therefore, effect of these parameters was evaluated on endoglucanase production by one factor at a time experiments. Initial studies by these experiments (data not shown) revealed that medium components did not have any significant effects on endoglucanase production whereas pH, moisture ratio, inoculum and fermentation time had significant effects on endoglucanase production. So the effects of pH, moisture ratio, inoculum and fermentation time were evaluated on the basis of endoglucanase production using PBD (Table 1). Among these variables, pH, moisture ratio and fermentation time were identified as the most significant and contributing variables for endoglucanase production (Figure 3) as *p*-values were 0.026, 0.027 and 0.007, respectively (Table 3). Based on these experiments, the values for the independent variables were set to enhance the endoglucanase production. For each run, the experimental responses and the predicted responses calculated from the regression equation (Equation 2) are presented in Table 5, which shows that the variables have strong effect on endoglucanase production. The results clearly indicated that moisture content had a positive effect to enhance endoglucanase production along with fermentation time. The influence of moisture content on fungal growth and product biosynthesis may be attributed to the impact of moisture on the physical properties of the solid substrates (Shah and Madamwar 2005). A higher than optimum moisture content causes gummy texture, decreased porosity, lower oxygen transfer, enhancement of the aerial mycelia and alternation in particle structure (Narahara et al. 1982).

On the basis of obtained experimental values, the statistical testing was performed using Minitab 16. The analysis of the model was tested by Student’s *t*-test and Fisher’s *F*-test. ANOVA of endoglucanase production showed that the response surface model was significant (*p* = 0.000), as shown in Table 7, contributed by linear and square terms of the regression model. A *p*-value below 0.05 indicates that the test parameter is significant. The fitted second-order response surface model as specified by Equation 2 for endoglucanase yield (*Y*) in coded process variables is as follows:

(3) $$Y=6.18746+0.01919x_{1}+0.72109x_{2}+1.60734x_{3}-0.02904{x_{1}}^{2}-0.97145{x_{2}}^{2}-0.99860{x_{3}}^{2}+0.07265x_{1}x_{2}-0.00176x_{1}x_{3}$$

**Table 7.** Analysis of variance for ln(EndoU/g)

Source	DF	Seq SS	Adj SS	Adj MS	*F*-Value	*p*-Value
Regression	8	40.8259	40.8259	5.1032	68.08	0.000
Linear	3	34.1034	34.1034	11.3678	151.65	0.000
pH	1	0.0029	0.0029	0.0029	0.04	0.849
Moisture ratio	1	18.5992	3.1199	3.1199	41.62	0.001
Fermentation time (h)	1	15.5013	15.5013	15.5013	206.79	0.000
Square	3	6.7014	6.7014	2.2338	29.80	0.001
pH*pH	1	0.0466	0.0031	0.0031	0.04	0.845
Moisture ratio*Moisture ratio	1	2.9728	3.4845	3.4845	46.48	0.000
Fermentation time (h)* Fermentation time (h)	1	3.6820	3.6820	3.6820	49.12	0.000
Interaction	2	0.0211	0.0211	0.0106	0.14	0.871
pH*Moisture ratio	1	0.0211	0.0211	0.0211	0.28	0.615
pH*Fermentation time (h)	1	0.0000	0.0000	0.0000	0.00	0.990
Residual Error	6	0.4498	0.4498	0.0750		
Lack-of-Fit	2	0.0789	0.0789	0.0394	0.43	0.680
Pure Error	4	0.3709	0.3709	0.0927		
Total	14	41.2757				

where *x_1_, x_2_* and *x_3_* are coded values of pH, moisture ratio and fermentation time, respectively. A comparison of the experimentally obtained values with the predicted values indicated that these data are in reasonable agreement as shown in Table 5. The parameter estimates and the corresponding *p*-values showed that two selected variables, that is, moisture ratio and fermentation time (0.001 and 0.000) had significant linear and square effect on endoglucanase yield (Table 6). In general, the corresponding coefficient is more significant when larger the magnitude of *t*-test statistic (*t*-value) and the smaller the value of *p* (Montgomery 1991). The *F*-test value for endoglucanase yield was 68.08, indicating that the developed model is highly significant as *p*-value is 0.000 (Table 6). Hence, the model on endoglucanase yield is true with almost no chance that an *F* value that large could have occurred due to noise.

The R^2^ value provides a measure of variability in the observed response values that can be explained by the experimental factors and their interactions. The coefficient of R^2^ was observed to be 98.91% for endoglucanase, which implies that only 1.09% of total variation was not explained by the model. The adjusted R^2^ was 97.46% which was in very good agreement with the predicted R^2^ value. Accordingly, as Table 5 shows, there is good agreement between the observed and predicted response values. The proximity of the adjusted R^2^ value to the predicted R^2^ clearly suggests that the model is very significant and can be used to predict the response.

**Figure 4.**
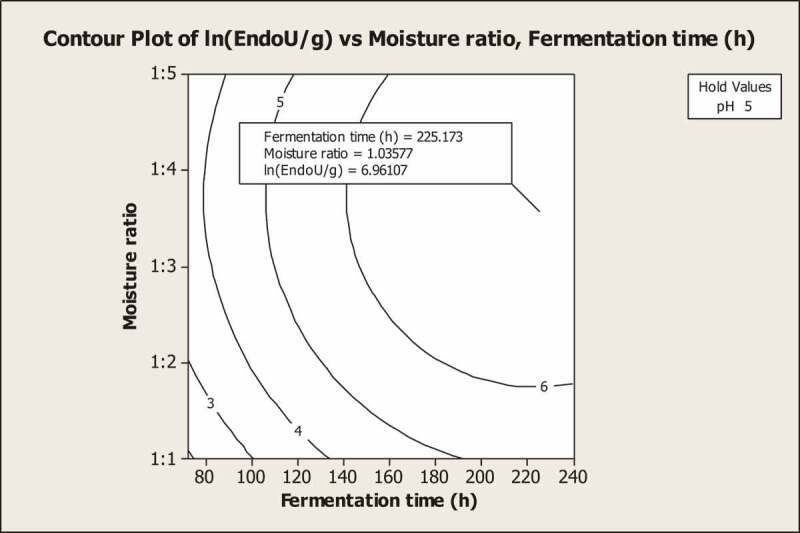
Contour plot showing interaction of moisture ratio and fermentation time on endoglucanase production at hold values of pH

In the present study, contour plots are used to graphically represent the interactive effect of two process variables on the response variable by holding one variable at a constant value (Figure 4). As pH did not have significant effects, it is held fixed and other process parameters, that is, moisture ratio and fermentation time are varied. The BBD fitted quadratic model given by equations (3) predicted that a maximum endoglucanase production 1054.76 U/g is achieved at moisture content of 1:3.5, pH 5 and 225.17 h of fermentation time.

A repeat fermentation for endoglucanase production was carried out under optimal conditions to validate the parameters predicted by the model. The endoglucanase production under optimised parameters, namely, moisture ratio 1:3.5, pH 5 and fermentation time of 225.17 h yielded endoglucanase activity of 1391.12 ± 21.13 U/g (173.89 ± 2.64 U/ml). This was significantly higher than the predicted value (1054.76 U/g) by the model. Thus, it is evident that the statistical optimisation increased endoglucanase production by 1.83-fold as compared to the initial production which was 760.29 ± 11.60 U/g (95.04 ± 5.55 U/ml). In addition, production of other cellulolytic and hemicellulolytic enzymes was also enhanced after statistical optimisation (Table 8). The production of endoglucanase by *Fomitopsis meliae* CFA 2 was higher or comparable with many of the reports on endoglucanase production by Ascomycetes and Basidiomycetes using SSF (Table 9). To the best of our knowledge, present study reports highest production of endoglucanase among the brown rot fungi reported. Most of the brown rot fungi are known to lack processive (exo-acting) cellulases, such as cellobiohydrolases, which are essential for cellulose hydrolysis. However, low level of cellobiohydrolase activity was observed in enzyme cocktail produced by *Fomitopsis meliae* CFA 2.

**Table 8.** Enzyme profile of crude enzyme produced by *Fomitopsis meliae* CFA 2 under optimised conditions

Enzymes	Enzyme activity (U/ml)	Enzyme activity (U/g)	Fold increase after optimization
Endoglucanase	173.89 ± 2.64	1391.12 ± 21.13	1.83
Filter paper activity	0.23 ± 0.00	1.80 ± 0.01	0.95
Cellobiohdyrolase	1.36 ± 0.05	10.88 ± 0.43	0.99
β-glucosidase	22.33 ± 0.18	178.64 ± 1.43	5.74
Endoxylanase	32.83 ± 0.06	262.64 ± 8.44	1.36
β-xylosidase	12.00 ± 0.07	96.00 ± 0.62	4.18
α-L-arabinofuranosidase	15.18 ± 0.85	121.44 ± 6.80	1.42
Mannanase	1.57 ± 0.02	12.55 ± 0.16	1.64

**Table 9.** Comparison of endoglucanase production by *Fomitopsis meliae* CFA 2 with other fungi under SSF

Fungi	Endoglucanase (U/g)	Substrate	Reference
*Trichoderma viride*	104.8	Banana peel	Das et al. 2011
*Trichoderma reesei* RUTC30	299.55	Wheat bran	Sukumaran et al. 2009
*Trichoderma harzianum* SNRS3	111.31	Rice straw	Rahnama et al. 2013
*Aspergillus niger* NS-2	310.0	Wheat bran	Bansal et al. 2012
*Aspergillus terreus*	141.29	Rice straw	Narra et al. 2014
*Aspergillus terreus*	581.0	Corn cob	Gao et al. 2008
*Aspergillus japonicus* URM5620	191.6	Castor Bean waste	Herculano et al. 2011
*Aspergillus fumigatus* Z5	526.3	Corn stover	Liu et al. 2011
*Aspergillus flavus* BS1	5,408.0	Tapioca flour in sawdust (teak wood)	Sajith et al. 2014
*Aspergillus tubingensis*	956.0	Sugarcane bagasse	Jain and Agrawal 2018
*Penicillium chrysogenum QML-2*	370.15	Corn stover/Wheat bran	Zhang and Sang 2012
*Fusarium oxysporum*	304.0	Corn stover	Panagiotou et al. 2003
*Neurospora crassa*	492.8	Wheat straw/bran	Dogaris et al. 2009
*Cladosporium cladosporioides* PAJ 03	88.51	Sugarcane bagasse/wheat bran	Marques et al. 2018
*Phomopsis stipata* SC 04	83.44		
*Phanerochaete chrysosporium*	188.66	Grass powder	Saratale et al. 2014
*Rhizopus stolonifer*	22,109.0	Coffee husk	Navya and Pushpa 2013
*Pleurotus dryinus*	401.0	wheat straw	Kachlishvili et al. 2006
*Myceliophthora heterothallica*	1,170.6	Sugarcane bagasse/wheat bran	Da Silva et al. 2016
*Microporus xanthopus* strain KA038	81.8	Green tea waste	Nguyen et al. 2019
*Latrunculia corticata*	450.0	Sugarcane bagasse/corn cob	El-Bondkly and El-Gendy 2012
*Lentinus tigrinus*	1,200.0	Wheat straw	Lechner and Papinutti 2006
*Thermoascus aurantiacus*	1,709.0	Wheat straw	Kalogeris et al. 2003
*Fomitopsis sp*. RCK2010	71.69	Wheat bran	Deswal et al. 2011
*Fomitopsis meliae* CFA 2	**1,391.12**	**Wheat bran**	**Present study**

Additionally, *Fomitopsis meliae* CFA 2 also released 17.24 ± 0.15 g/L of reducing sugars (6.90 ± 0.14 g/L glucose and 8.33 ± 0.13 g/L xylose) in crude enzyme extract. This higher sugar release is presumed to derive from the effects of early-stage brown rot degradation mechanism. In which, the cellulose and hemicellulose components of cell wall are extensively and rapidly depolymerised. It has been suggested that this process is not under enzymatic control as the depolymerisation is faster than the utilisation of degradation products by the brown rot fungi (Ray et al. 2010). Moreover, 1.587 ± 0.014 g/L (~12.59 mM) of oxalic acid was also detected in crude enzyme extract. Secretion of oxalic acid is a characteristic feature of brown rot fungi for initiation of Fenton reaction chemistry required for degradation of cellulose-hemicellulose in addition to carbohydrate active enzyme. Release of oxalic acid results in lowering of pH and thereby chelation of Fe^3+^ in the vicinity of fungal hyphae and initiating Fenton chemistry (Shah et al. 2018). Extracellular Fe^3+^ reducing activity produced by *Fomitopsis meliae* CFA 2 was analysed from the crude extract and was found to be 3.72 ± 0.02 mM. These findings clearly indicated that *Fomitopsis meliae* CFA 2 presented noteworthy cellulases and hemicellulases activities together with substantial accumulation of oxalic acid and release of Fe^3+^ reducing activity.

### Effect of temperature and pH on endoglucanase activity

**Figure 5.**
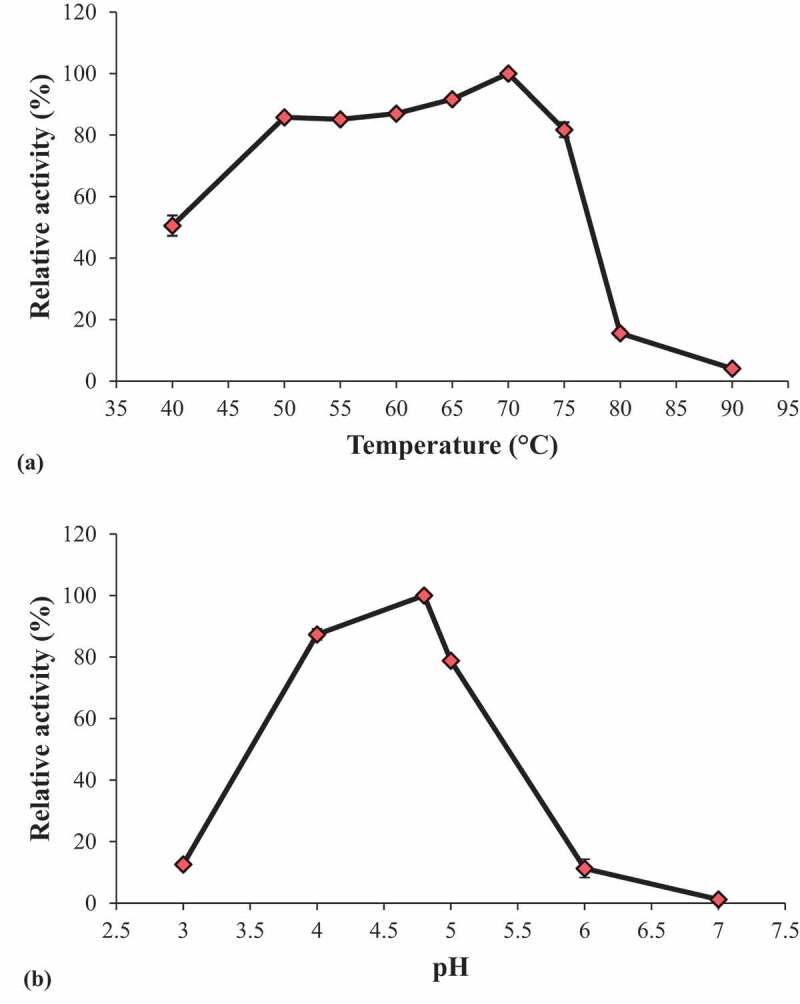
Effect of temperature (a) and pH (b) on endoglucanase activity

The influence of temperature on endoglucanase activity was evaluated in the range of 40°C to 90°C. The results revealed that the optimum temperature for endoglucanase activity was 70°C (Figure 5a). The enzyme was remarkably active at 50°C, 55°C, 60°C and 75°C with loss of only 14.24%, 14.86%, 13%, 8.36% and 18.26% activity, respectively. Endoglucanase from thermophilic fungus *Myceliophthora heterothallica* was reported to be active at 60°C (Da Silva et al. 2016). The endoglucanase activity at various pH (3.0 to 7.0) was measured using carboxymethyl cellulose as a substrate at 55°C. The optimum pH for endoglucanase activity was found to be at 4.8 (Figure 5b). Endoglucanase activity was reduced by 12.63% and 21.17% at pH 4.0 and 5.0, respectively. Most of the endoglucanase exhibited its optimum activity between the ranges of pH 4.8 to 5.0.

### Effect of temperature on endoglucanase stability

**Figure 6.**
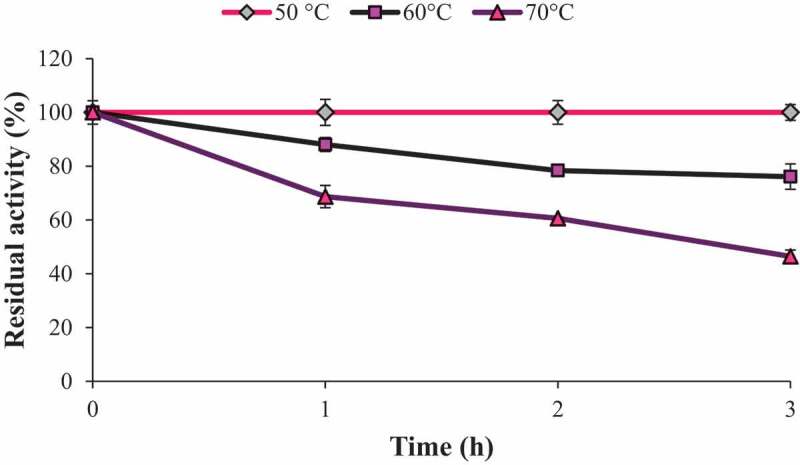
Effect of temperature on endoglucanase stability

The endoglucanase from *Fomitopsis meliae* CFA 2 was found to be highly stable at 50°C up to 3 h. At 60 and 70°C, residual enzyme activity was 76.12% and 46.46% after 3 h, respectively (Figure 6). Crude endoglucanase was more stable than endoglucanase from *Myceliophthora heterothallica* (da Silva et al. 2016) and *Aspergillus japonicus* URM5620 (Herculano et al. 2011). Endoglucanase from thermophilic fungus *Myceliophthora heterothallica* lost around 80% activity after 1 h at 70°C whereas *Aspergillus japonicus* was stable at 50°C up to 90 min. Picart et al. (2007), showed that endoglucanase from *Penicillium sp*. remained stable at 60°C for at least 3 h, while at 65°C the enzyme lost 75% of its activity after 1 h of incubation. Higher thermostability is preferable for application of endoglucanase in biomass saccharification.

### Evaluation of crude enzyme for enzymatic saccharification of agro-residues

**Figure 7.**
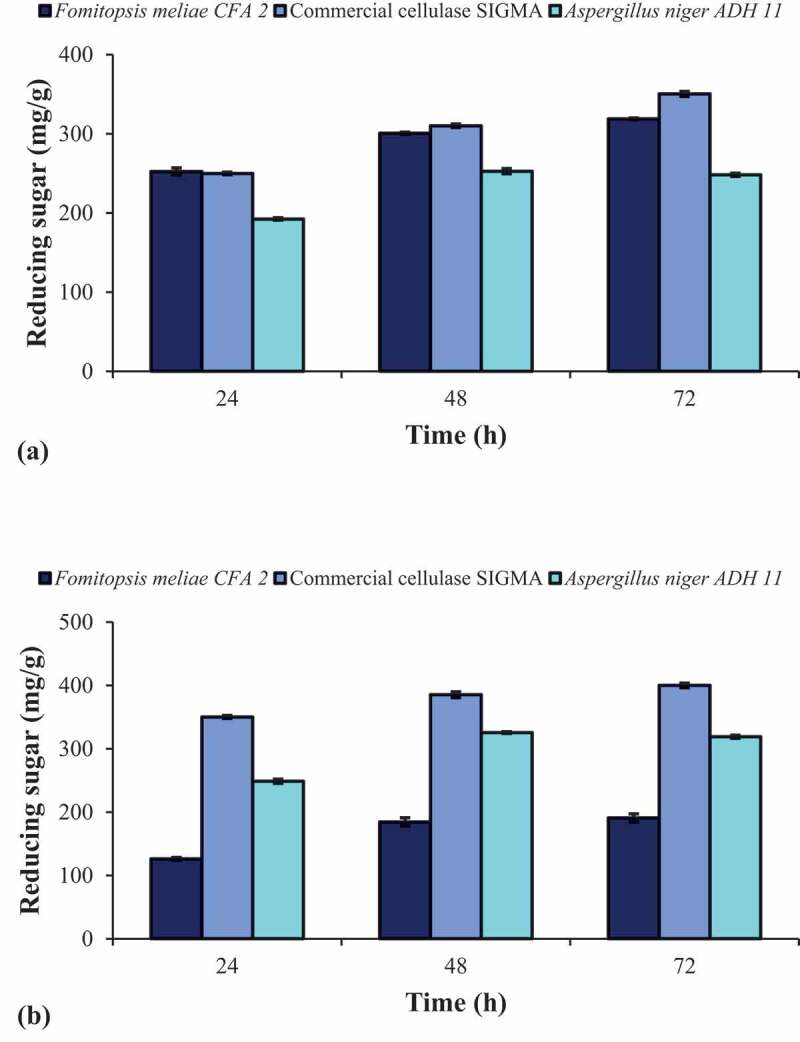
Profile of enzymatic saccharification of alkali-treated (a) rice straw and (b) wheat straw using 5 FPU/g of crude enzyme from *Fomitopsis meliae* CFA 2, commercial cellulase SIGMA and crude enyme from *Aspergillus niger* ADH 11

To understand the effects of pretreatment on wheat straw and rice straw, the composition of untreated and alkali-treated agro-residues was checked. The cellulose, hemicellulose and lignin contents of untreated wheat straw was 43.81%, 36.00% and 22.2%, respectively, whereas pretreated wheat straw was 71.49%, 15.07% and 5.39%, respectively. The cellulose, hemicellulose and lignin contents of untreated rice straw was 39.00%, 32.20% and 12.30%, respectively, whereas pretreated rice straw was 74.70%, 20.60% and 2.30%, respectively. In both the cases, pretreatment was resulted in enrichment of cellulose content and high level of delignification. The hydrolytic efficacy of crude enzyme was evaluated by enzymatic saccharification of alkali-treated wheat straw and rice straw and compared with the cellulases from *Aspergillus niger* ADH-11 and SIGMA. The commercial cellulase SIGMA and crude enzyme from *Aspergillus niger* ADH-11 was previously reported to be effective in saccharification of MAA treated wheat straw (Patel et al. 2017). The time course profile of enzymatic saccharification using 5 FPU/g of enzyme dose is shown in Figure 7. The maximum 318.8 mg/g of reducing sugars were released from alkali-treated rice straw (Figure 7a) whereas 190.8 mg/g of reducing sugars were released from alkali-treated wheat straw (Figure 7b) after 72 h of hydrolysis period using crude enzyme from *Fomitopsis meliae* CFA 2. The saccharification yield was found to be 38.41% and 24.02% after enzymatic saccharification of rice straw and wheat straw using crude enzyme from *Fomitopsis meliae* CFA 2. As compared to SIGMA cellulase, reducing sugar yield is lesser from alkali-treated wheat straw and rice straw, which may due to lesser titre of exoglucanase. Overall, it can be inferred that the hydrolytic efficacy of crude enzyme of *Fomitopsis meliae* CFA 2 is comparable with the hydrolytic efficacy of commercial enzyme SIGMA and crude enzyme from *Aspergillus niger* ADH-11 in case of rice straw. However, further studies are needed to establish the efficacy of crude enzyme of *Fomitopsis meliae* CFA 2 in biomass saccharification.

## Conclusions

The present investigation showed that newly isolated *Fomitopsis meliae* CFA 2 is a potential endoglucanase producer. The statistical optimisation for endoglucanase production by SSF was highly advantageous as endoglucanase production was enhanced by 1.83-fold. The production of other cellulolytic and hemicellulolytic enzymes was also enhanced simultaneously after statistical optimisation. The findings revealed the ability of brown rot fungi *Fomitopsis meliae* CFA 2 to produce multi-enzyme cocktail which can be suitable for biomass saccharification.
